# Early and complete vitrectomy versus tap and inject in acute post cataract surgery endophthalmitis presenting with hand motion vision; a quasi-experimental study

**DOI:** 10.1186/s12886-022-02247-8

**Published:** 2022-01-08

**Authors:** Seyed Ali Tabatabaei, Soran Aminzade, Aliasghar Ahmadraji, Mohammad Soleimani, Bahram Bohrani Sefidan, Abolfazl Kasaee, Kasra Cheraqpour

**Affiliations:** Eye Research Center, Farabi Eye Hospital, Tehran University of Medical Sciences, Tehran, 1336616351 Iran

**Keywords:** Endophthalmitis, Endophthalmitis vitrectomy study, EVS, Cataract surgery, Vitrectomy

## Abstract

**Background:**

Based on endophthalmitis vitrectomy study, intravitreal injection of antibiotics is preferred for initial management of cases of acute post cataract surgery endophthalmitis (APCE) with presenting vision of hand motions (HM). This study aimed to compare outcomes of early and complete vitrectomy (VIT) and vitreous tap and antibiotic injection (T&I) in cases of APCE presented with vision of HM.

**Methods:**

In this prospective study, cases of APCE with vision of HM between 2018 and 2020 were enrolled. According to the time of presentation, the patients were arranged into two groups (VIT vs. T&I). Demographic data, elapsed time to developing endophthalmitis, past medical history, microbiology results, complications, and final visual acuity were recorded and analyzed.

**Results:**

Seventy-six eyes of 76 patients were enrolled. Fifty-three eyes underwent T&I and twenty-three were arranged into the VIT group. Past medical history of 34.2% of patients was significant for diabetes mellitus. There was a statistically significant lower logMAR in VIT group compared to T&I group (diff = 0.14, 95% CI: 0.04 to 0.24, *P*-value = 0.007). The comparison of the diabetic and non-diabetic patients in both groups showed that the visual outcome was better in non-diabetic cases compared to the diabetic subjects. There was no statistically significant difference between the diabetic and non-diabetic groups regarding the superiority of procedure.

**Conclusion:**

Based on our results, we could recommend that it’s maybe better to do early and complete vitrectomy as the initial management of APCE with the vision of HM. Past medical history of diabetes mellitus is not a determining factor for choosing initial management between vitrectomy and antibiotic injection.

## Background

Nowadays cataract surgery is one the most common ocular surgeries worldwide, which can significantly improve the quality of life. Endophthalmitis is the most disastrous complication of cataract extraction leading to severe vision loss even evisceration of the eye [1, 2].

Since 1995, the endophthalmitis vitrectomy study (EVS) has been become the most acceptable treatment strategy for vitreoretinal surgeons to manage patients of post cataract surgery endophthalmitis [3]. This study showed that outcomes of vitrectomy or tap and intravitreal injection of antibiotics (T&I) are equal in the visual acuities (VA) of hand motions (HM) or better whereas in vision of light perception (LP) vitrectomy is superior. Despite all of the improvements and innovations in the surgical techniques and devices, this approach remained popular and favorable among a large number of ophthalmologists [4, 5]. Great technical improvements have been led to thoroughly removing of vitreous and inflammatory debris, safer induction of posterior vitreous detachment (PVD), and better peripheral vitreous shaving. It’s of note EVS is still the only prospective randomized study to manage acute endophthalmitis following cataract surgery.

The goal of our study is to compare between outcomes of early and complete vitrectomy (VIT) and T&I in cases of acute post cataract surgery endophthalmitis presented with vision of hand motions.

## Material and methods

This prospective study was conducted at the emergency department of Farabi eye hospital, Tehran, Iran. Cases of acute post cataract surgery endophthalmitis with vision of HM between April 2018 and July 2020 were enrolled. All of the included patients had history of uncomplicated phacoemulsification with clear cornea incision. Patients with endophthalmitis due to other surgical procedures, history of prior visual loss such as retinal detachment (RD), glaucoma, diabetic retinopathy of any stages (based on examination of the fellow eye and the same eye after clearance of media), positive ocular history for vitreoretinal surgeries, non-HM vision, and also posttraumatic or endogenous endophthalmitis were excluded from the study. Furthermore, history of intracameral antibiotic injection or surgical complications such as vitreous loss was considered an exclusion criteria.

Based on the EVS method for detecting vision, we examined all APCE patients to include only the patients with HM vision. The best-corrected vision was determined using the Snellen acuity chart at 4 m; if no letters could be read, the test was done at 1 m and then the vision was tested for the ability to count fingers. If the patient could count fingers or more, the patient was excluded from the study. Otherwise HM vision was tested using a light source directed from behind the patient accompanied by occlusion of patient’s fellow eye. The examiner’s hand was either stationary or moved in a horizontal or vertical direction at a distance of 60 cm from the eye. The HM vision test was repeated five times and if the patient could recognize the hand movement and alignment on at least four of the presentations, the HM visual acuity was considered and the patient was included. If the hand motions could not be detected, vision of LP was tested and the patient was excluded from the study.

Acute endophthalmitis was defined as developing symptoms of pain and decreased vision and clinical findings of endophthalmitis such as hypopyon, vitritis, and poor red reflex within 6 weeks after cataract surgery. Diagnosis was confirmed by an experienced vitreoretinal surgeon. Surgeries were done immediately within 3 h of patient admission to our center. Patients were divided into two groups: in one group primary pars plana vitrectomy (PPV) was done by an expert vitreoretinal surgeon and patients of the other group underwent anterior chamber (AC) and vitreous tap and intravitreal injection of antibiotics. Secondary vitrectomy was defined as the need for vitrectomy or re-vitrectomy (in PPV group) within 2 weeks of initial procedure. A classic randomization was almost impossible due to poor access to an expert vitreoretinal surgeon during midnight hours. Hence, based on the time of presentation to the hospital the patients were arranged into two groups; the patients who were admitted in the time period of 6:00 am until 9:00 pm scheduled for immediate pars plana vitrectomy by an expert surgeon and the rest (patients in time of 9:00 pm to 6:00 am) underwent for AC and vitreous tap and intravitreal injection of antibiotics.

The goal of vitrectomy was removing infective materials as much as possible without inducing iatrogenic damage. Induction of PVD was tried for all of the cases with intact vitreoretinal interface. After core vitrectomy, brief shaving of peripheral vitreous was done. At the end of surgery 1 mg vancomycin and 2.2 mg ceftazidime were injected into the vitreous. In the T&I group after vitreous tap and sampling, 1 mg vancomycin and 2.2 mg ceftazidime were injected into vitreous. Patients of both groups were admitted to the emergency ward of hospital for receiving systemic and fortified antibiotics (vancomycin and ceftazidime), corticosteroid (betamethasone) eye drop, cycloplegic agents (mostly homatropine) and also close observation to assess the signs of improvement or deterioration. If serial examination (every 6 h) was suggestive for worsening or lack of improvement, decision for additional action would be made. Prone or lateral decubitus position was advised to all of the patients to prevent from macular toxicity of inflammation or injected drugs. Minimum of follow-up time was 6 months and the patients who did not complete their follow- up visits were excluded.

Demographic data, elapsed time to developing endophthalmitis, type of primary and occasionally subsequent intervention, past medical history such as diabetes mellitus (DM), microbiology results, complications such as RD, and final best-corrected visual acuity (BCVA) in logMAR scale were recorded on an excel spreadsheet. To present data we used mean, standard deviation, median and range, and frequency and percentage. To compare groups in baseline we used t-test, Mann-Whitney test, Chi-square and Fisher exact test. In order to compare the trend of VA in two groups during the follow-up visits, we used linear mixed model. Another linear mixed model was used to adjust for the effect of possible confounders. All statistical analysis performed by SPSS (IBM Corp. Released 2017. IBM SPSS Statistics for Windows, Version 25.0. Armonk, NY: IBM Corp.). *P*-value less than 0.05 considered statistically significant.

This research was undertaken in accordance with the Declaration of Helsinki and confirmed by Farabi Eye Hospital Institutional Review Board (IR.TUMS.FARABIH.REC.1398.006). Written informed consent was obtained from all participants.

## Results

Seventy-six eyes of 76 patients were enrolled in the study. Forty patients were male and thirty-six individuals were female. Fifty-three eyes underwent T&I and twenty-three were arranged into the VIT group. Past medical history of 34.2% of patients was significant for DM (26 individuals). Also, 26 patients mentioned history of hypertension. The mean time of presentation after developing of endophthalmitis was 6.7 ± 3.5 and 5.2 ± 2.3 days for T&I and VIT groups, respectively. Initial microbiological studies showed 52.8% and 34.8% negative culture for T&I and VIT groups, respectively. Among the 26 diabetic patients, 12 eyes had negative culture result, 11 eyes showed growth of *Staphylococcus epidermidis*, and in 3 eyes culture was positive for *Staphylococcus aureus*. In the follow-up visits, 1.3% (1 patient), 2.6% (2 patients), and 1.3% (1 patient) of patients were complicated with RD, pseudophakik bullous keratopathy, and necrotizing scleritis, respectively, all occurred in T&I group. Secondary vitrectomy was done for 21 patients (39.6%) of the T&I group and one patient (4.3%) of VIT group. Among the 21 patients, 7of them (33.3%) underwent secondary vitrectomy the day after vitreous tap and antibiotic injection, 10 patients (47.6%) between day two and seven and 4 patients (19%) between day eight and fourteen. Descriptive data are summarized in Table 1.

**Table 1:** Descriptive analysis († Based on t-test, ‡ Based on Mann-Whitney test, * Based on Chi-square test, ** Based on Fihser exact test)

Parameter		Group		***P***-val.
		T&I	VIT	
**Age (year)**	Mean ± SD	64 ± 11	66 ± 8	0.524†
	Median (range)	64 (38 to 88)	64 (51 to 80)	
**Onset time (day)**	Mean ± SD	6.7 ± 3.5	5.2 ± 2.3	0.202‡
	Median (range)	5 (2 to 15)	5 (3 to 10)	
**Gender**	Male	27 (50.9%)	13 (56.5%)	0.655*
	Female	26 (49.1%)	10 (43.5%)	
**Diabetes mellitus**	Positive	17 (32.1%)	9 (39.1%)	0.551*
	Negative	36 (67.9%)	14 (60.9%)	
**Hypertension**	Positive	17 (32.1%)	9 (39.1%)	0.551*
	Negative	36 (67.9%)	14 (60.9%)	
**Culture**	Negative	28 (52.8%)	8 (34.8%)	0.021**
	*Staphylococcus epidermidis*	23 (43.4%)	9 (39.1%)	
	*Staphylococcus aureus*	2 (3.8%)	5 (21.7%)	
	*Streptococcus pneumoniae*	0 (0.0%)	1 (4.3%)	
**Complication**	Negative	49 (92.5%)	23 (100.0%)	> 0.99**
	Retinal detachment	1 (1.9%)	0 (0.0%)	
	Pseudophakik bullous keratopathy	2 (3.8%)	0 (0.0%)	
	Necrotizing scleritis	1 (1.9%)	0 (0.0%)	
**Secondary vitrectomy**	Negative	32 (60.3%)	22 (95.6%)	0.042**
	Secondary vitrectomy	21 (39.6%)	1 (4.3%)	

Table 2 shows BCVA (logMAR scale) of presentation, 1st, 3rd, and 6th month of follow-up visit in different groups of study

**Table 2:** Presenting and follow-up BCVA in both groups

	Group	
	T&I	VIT
BCVA0	2.4 ± 0	2.4 ± 0
BCVA1	1.3 ± 0.4	1.3 ± 0.4
BCVA3	1 ± 0.3	0.9 ± 0.4
BCVA6	0.8 ± 0.3	0.7 ± 0.4

Linear mixed model after adjustment of DM status showed a statistically significant lower logMAR in VIT group in comparison to T&I group (diff = 0.14, 95% CI: 0.04 to 0.24, *P*-value = 0.007).

Also, linear mixed model showed that there was a statistically significant lower logMAR in VIT group in comparison to T&I in non-diabetic cases (diff = 0.16, 95% CI: 0.03 to 0.30, *P*-value = 0.017). However, this analysis showed that the difference of the two groups were not statistically significant in diabetic group after adjustment of the baseline values (diff = 0.11, 95% CI: − 0.03 to 0.25, *P*-value = 0.135) (Table 3).

**Table 3:** Linear mixed model after adjustment of baseline BCVA to compare logMAR of vitrectomy and antibiotic groups in non-diabetic and diabetic patients

	Diff.	*P*-val.	95% Confidence Interval	
			Lower Bound	Upper Bound
Diabetic	0.11	0.135	−0.03	0.25
Non-diabetic	0.16	0.017	0.03	0.30

The comparison of the diabetic and non-diabetic patients in both groups showed that the VA was better in non-diabetic cases compared to the diabetic subjects (diff = 0.29, *P*-value < 0.001 in T&I group, diff = 0.37, *P*-value < 0.001 in VIT group). Based on interaction analysis with a linear mixed model, no statistically significant difference in VA outcome was detected between the diabetic and non-diabetic groups regarding the superiority of the procedure (*P* = 0.542) (Table 4).

**Table 4:** The comparison of the DM positive and non-positive groups in both groups

	Diff.	*P*-val.	95% Confidence Interval	
			Lower Bound	Upper Bound
T&I	0.29	< 0.001	0.18	0.41
VIT	0.37	< 0.001	0.19	0.54

On the issue of relationship between culture result and final BCVA, after adjustment of DM status, the subjects with negative culture had a lower logMAR compared to positive culture subjects (diff = 0.16, 95% CI: 0.07 to 0.25, *P* = 0.001).

Table 5 shows comparison of visual outcome in silicone oil (SO) filled eyes with non-silicone filled vitrectomized eyes. According to the analysis there is a significant better visual acuity in non-silicone filled eyes. It is of note that BCVA of sixth month of follow-up was measured after SO removal. There were 4 SO filled eyes (17.3%) in VIT group and 3 SO filled eyes (14.2%) in T&I group.

**Table 5:** Comparison of BCVA in silicone filled and non-silicone filled eyes. (‡ Based on Mann-Whitney test)

	Silicone				*P*‡
	SO Filled		Non-filled		
	Mean	SD	Mean	SD	
BCVA1	1.7	0.2	1.3	0.4	0.008
BCVA3	1.5	0.3	1.0	0.4	0.006
BCVA6	1.5	0.5	0.7	0.3	< 0.001

## Discussion

Our study on 76 patients with APCE presented with a vision of HM showed a significantly better vision in 6 months follow-up in VIT group compared to T&I group. Furthermore, VA was better in non-diabetic cases than the diabetic subjects in both groups.

The mainstay of APCE management has been EVS since 1995. EVS was the only prospective RCT conducted in over than 20 centers in USA in the 1990s. Four hundred-twenty patients with acute post cataract surgery endophthalmitis were studied in a 4-year period to clarify the effect of intravenous antibiotics and also differences between outcomes of vitrectomy and intravitreal injection of antibiotics. In patients with initial vision of LP, outcomes of vitrectomy were significantly better than intravitreal antibiotics meanwhile no statistically difference between vitrectomy and intravitreal antibiotics was reported in initial VA of HM or better. Furthermore, employing of intravenous antibiotics did not result in better visual outcome [3].

Endophthalmitis vitrectomy study has been shown that final visual acuity of approximately 50% and 25% of patients with acute post cataract surgery endophthalmitis is < 20/40 and ≤ 20/200, respectively. In the current century, advances in devices such as vitrectomy probes or microscopes and surgical techniques could revolutionize these poor visual outcomes but this event did not happen in real and all of the recent studies reported the same or even worse outcomes than EVS [6]. Remarkable number of ophthalmologists is still bounded to EVS, which recommend intravitreal injection of antibiotics in eyes with vision of HM or better [7, 8]. This approach may deprive the patients from advantages of vitrectomy. Unlike EVS, which the primary aim of core vitrectomy was gathering specimens for microbiological evaluation but not clean up the infection source, nowadays new technology has been facilitated removing of pyogenic materials of cortical vitreous on the retinal surface without iatrogenic damages [9, 10]. Several studies have been shown that retinal damages can be continued even in condition of undetectable counts of bacteria supporting the prominent role of remained toxins, inflammation, and the host’s immune system [6]. Hence, removing of infection source can be considered the advantage of vitrectomy over antibiotic injection.

Previously, it has been established that intravitreal injection of antibiotics is not able to sterilize the vitreous cavity, which can be related to biofilm formation by microorganism such *Pseudomonas aeruginosa* and *Staphylococcal* spp. [11–14]. Crosby et al. [15] reported that viable bacteria can be found after tap and intravitreal injection of antibiotics but not vitrectomy. However, Leung et al. [13] detected positive cultures even in their vitrectomy cases. This incoherency in results can be interpreted based on the different times of studies; Crosby et al. [15] enrolled their cases of endophthalmitis since 2007, when development of new techniques and devices made complete removing of infection source easier. On the other hand Leung et al. [13] performed a retrospective study on cases of over last three decades. It seems possibility of taking secondary action is less in cases of primary vitrectomy in comparison to initial treatment with intravitreal injection of antibiotics. This finding can be rationalized by the presence of viable bacteria despite intravitreal injection of antibiotics. In a study, 68% and 26% of additional procedures were reported for group of intravitreal antibiotic injection and vitrectomy, respectively [15]. However, in our study the rate of additional procedure (secondary vitrectomy) was 39.6% for T&I group and 4.3% for VIT group. This difference demonstrates the important role of a complete vitrectomy and pus clearance in controlling disease progression and decreasing the need to more procedure. Breakdown products from bacterial cell walls may continue to incite inflammatory response, so removal of these materials will limit ongoing retinal damage in cases with resistance to initial antibiotic injection [16].

In the EVS about 8 % of eyes experienced retinal detachment (7.8% of vitrectomized eyes and 9% of patients in group of tap and intravitreal injection). It seems that the endophthalmitis-related RDs are developed due to retinal necrosis and the role of iatrogenic breaks is less significant. Necrotic areas are more common where pus is accumulated by gravity. Remove of purulent materials through a complete vitrectomy followed by intravitreal injection of antibiotics may decrease the risk of developing retinal tears on necrotic retina [6]. Only one case (1.9%) experienced retinal detachment in our study. This low rate can be related to the prospective nature and short duration of follow-up time in our study whereas previous studies had a retrospective design with longer period of follow-up time. It should be mentioned that we used silicone oil in cases with retinal necrosis that were in significant risk for developing retinal breaks and subsequent retinal detachment. Furthermore, some of the vitreoretinal surgeons believe that silicon oil may have bacteriostatic and bactericidal properties.

Some studies have reported that culture-negative eyes may be required additional actions less than culture-positive eyes- [17]. Culture-negative report in microbiological studies may be representative for lower bacterial load or virulence. It should be mentioned that possibility of postoperative uveitis or toxic anterior segment syndrome (TASS) is considerable in this condition. However, clinical response to antibiotics is a differentiating factor [18]. It has been shown that the most common detected organisms in culture-positive endophthalmitis cases following cataract surgery are the Gram-positive organisms, which are responsible for 95% of culture-positive results. Gram-positive bacteria in a decreasing order of frequency include *coagulase-negative Staphylococcus (CoNS)*, *Staphylococcus aureus*, *Streptococcus* species, and *Enterococcus* species. Gram-negative species account for about 5–6% of cultures and include *Pseudomonas*, *Proteus*, and *Haemophilus Influenzae*. Interestingly, our study was not compatible with this finding since all of our positive cultures were Gram- positive cocci [17, 19–22]. One reason was that the APCE with Gram-negative cultures is more prone to present with worse VA. Visual outcomes of *CoNS* are remarkably better than *Streptococcal* cases of endophthalmitis. In the EVS, number of patients with *CoNS* endophthalmitis who gained final vision of 20/100 or better was about 3 times more than *Streptococcal* endophthalmitis [23]. This finding can be related to the more production of exotoxins by *Streptococcal* species [24]. In a study by Lee et al. [18], culture-positive cases of post operative endophthalmitis had worse visual outcomes and higher rates of complication with RD compared to negative-culture eyes. Our results were in the same direction.

Complete vitrectomy was introduced as the gold standard procedure for management of post operative endophthalmitis by guideline of ESCRS in 2013. Lowering the chance of additional procedures and removing the infective materials from vitreous can be considered the basic advantages of this recommendation. In opinion of some of vitreoretinal experts, decision making on the type of management in cases of postoperative endophthalmitis should not be limited to initial vision but also clinical findings such as inability to see the fundus, rapid progressive vision loss, and possibility of infection by virulent organisms like *streptococci* can be persuasive for choosing vitrectomy [25].

At the time being, endophthalmitis vitrectomy study is the only multicenter, prospective RCT to compare the outcome of surgical (vitrectomy) and non-surgical management (intravitreal antibiotics) of acute endophthalmitis following cataract surgery. To the best of our knowledge, our study is the second study with prospective nature on the management of acute post cataract surgery endophthalmitis. In our study only patients with presenting vision of HM were enrolled and unlike the EVS patient with better or worse visions were excluded. So, the difference in the sample size of our study and EVS is related to inclusion criteria since EVS has been included 420 eyes with variable presenting visions meanwhile in our study 76 eyes with vision of HM were enrolled. Regarding the issue of randomization, one of the weak points of our study was lack of classic randomization. However, arranging the patients into two groups based on the presentation time (day or night) can be considered a type of allocation and randomization. On the other hand, the second weak point is our culture-proven results; all positive culture patients were Gram-positive. However, limiting the patients based on visual acuity of hand motion maybe is the cause since patients with Gram-negative cultures usually present with LP or NLP.

The main purpose of our study was the investigation on possible effectiveness and advantage of vitrectomy over the intravitreal injection of antibiotics. It should be mentioned EVS is still a so valuable and reliable study for decision making even for authors of this study. Our study was not conducted to question the EVS. The goal of our study was to propose and strengthen the usage of vitrectomy in APCE cases as a relatively novel idea which can be a basic report for performing more studies in the future. The authors have no claim regarding the generalization of the results of this study and more studies are required for supporting of this idea.

## Conclusion

Based on our findings, we could recommendthat it’s maybe better to perform early and complete vitrectomy as the initial plan for the management of cases of acute post cataract surgery endophthalmitis with a vision of hand motions. Solely past medical history of diabetes mellitus is not a determining factor for choosing initial management between vitrectomy or tap and antibiotic injection. However, diabetic patients experience worse outcomes compared to healthy individuals.

## Data Availability

The data is available from the corresponding author on reasonable request.

## Abbreviations

APCE: Acute post cataract surgery endophthalmitis
HM: Hand motions
T&I: Tap and antibiotic injection
VIT: Early and complete vitrectomy
EVS: Endophthalmitis vitrectomy study
VA: Visual acuity
LP: Light perception
PVD: Posterior vitreous detachment
PPV: Pars plana vitrectomy
AC: Anterior chamber
BCVA: Best-corrected visual acuity
DM: Diabetes mellitus
SO: Silicone oil
RD: Retinal detachment
TASS: Toxic anterior segment syndrome
